# Trends in Private Equity Acquisition of Orthopaedic Surgery Practices in the United States

**DOI:** 10.5435/JAAOSGlobal-D-21-00162

**Published:** 2021-12-20

**Authors:** Christopher Mikhail, Dhruv Shankar, Amir Taree, Kush Mody, Joseph Barbera, Jeffrey Okewunmi, Samuel Cho, Shawn Anthony

**Affiliations:** From the Department of Orthopaedic Surgery, Icahn School of Medicine, New York, NY.

## Abstract

**Background:**

Independent orthopaedic practices in the United States have become attractive targets for acquisition by hospital systems and private equity (PE) firms because of the increasing demand for outpatient surgery. Consolidation in this market will have notable effects on the delivery and cost of orthopaedic services. In this study, we identified major trends in orthopaedic practice acquisitions over the past decade.

**Methods:**

A list of acquisition deals between 2010 and 2019 was compiled from four business databases: S&P Capital IQ, CB Insights, Thomson ONE, and Zephyr. Deals were categorized as PE-backed or not PE-backed. Headquarter locations of the buying and selling companies and transaction value were obtained for each deal when available.

**Results:**

A total of 68 deals were obtained of which 5 (7.4%) were PE-backed. The buyer and seller were located in the same state in 50 (73.5%) of the deals. Transaction values were available for only four deals ranging from $2.52 million to $35 million.

**Conclusion:**

Our results suggest that consolidation of orthopaedic practices from 2010 to 2019 was driven by large healthcare entities rather than PE firms. Furthermore, intrastate acquisitions were markedly more common than interstate acquisitions, possibly because of greater legal feasibility and ease of clinical integration.

Physician practice consolidation has become a growing trend over the past decade. Although considerable variation exists across different specialties, consolidation of physician practices by hospitals rose across all specialties from 2007 to 2017, particularly in medical and surgical subspecialties.^1^ An increasing number of these consolidations are products of private equity (PE)–backed acquisitions. From 2010 to 2017, the number of PE deals involving healthcare-related companies increased by 48% and the value of these deals increased by 187%, reaching $42.6 billion. Of note, in 2017, healthcare deals made up 18% of all PE deals worldwide.^2^

Similar to trends seen in dermatology, ophthalmology, urology, and gastroenterology orthopaedic practices have caught the attention of investors seeking to bolster economic value by consolidating practices.^2-4^ Acquisition and consolidation of practices produce economies of scale, increasing cost-effectiveness, revenue, and market share.^5^ From the clinical perspective, consolidation increases both the coordination of care and stability of physician networks.^6^

Expenditures related to orthopaedic care are projected to grow rapidly to support an aging population.^7^ Coupled with a shift toward procedures done in outpatient or ambulatory surgery settings, orthopaedic practices are ripe for consolidation.^8^ Orthopaedic clinics are projected to increase the provision of ancillary services such as radiograph, MRI, physical therapy, occupational therapy, and durable medical equipment. Growth in the demand for these services will produce financial pressures that can be alleviated by scale.^9^ Financial support can offset this concern while growing practices and improving infrastructure in existing practices.^10^ Health systems, payers, PE investors, and large medical groups are suitable investors in physician practices, creating opportunities for vertical (acquisition by hospitals and health systems) and horizontal consolidation (acquisition by other practices).^6^

Currently, studies on investment trends in orthopaedic surgery are yet to be published. Our study aims to identify recent trends in acquisitions and consolidation of orthopaedic practices in the United States using a reproducible method. Beginning from transactions in May 2012, we intend to quantify the number of practices acquired in addition to mapping these trends geographically. Finally, the study will discuss the possible future of acquisitions given the uncertainty of the economic climate due to the COVID-19 pandemic.

## Methods

Records of acquisition deals were obtained from four business databases: S&P Capital IQ, CB Insights, Thomson ONE, and Zephyr (Table 1). The databases were queried for all deals closed between January 1, 2010, and December 31, 2019, that involved an acquisition of a full or majority stake in an independent single-specialty practice that provided orthopaedic services and was located in the United States. Practice types included, but were not limited to, solo practices, group practices, physician groups, ambulatory surgery centers (ASCs), and hospitals. An acquisition was only included in the analysis if the acquired practice provided orthopaedic medical and/or surgical care as its primary service. Acquisitions of practices that specialized in joint arthroplasty or spine care were included if the main care providers at the practice were orthopedists or orthopaedic surgeons. Any acquisition in which the practice sold was a multispecialty clinic, neurosurgical spine clinic, physical therapy clinic, occupational therapy clinic, medical device company, physician staffing company or otherwise not primarily an orthopaedic medical/surgical practice was excluded from the analysis. Mergers and acquisitions of minority stake were excluded as well.

**Table 1:** Search Criteria for Each Database Used to Obtain Acquisition Records

Database	Search Criteria
S&P Capital IQ	• All transactions closed date (“January 1, 2010, to December 31, 2019”) • Geographic location of target/issuer (“United States of America”) • Industry classifications of target/issuer (“orthopaedic services”) • Transaction types (“merger/acquisition”)
CB Insights	• Advanced search, company search, industry, and geography • Geography (“United States, North America”) • Company industries (“health care” and “medical facilities and services”) • Company attributes and keywords (“orthopedics,” “orthopaedics,” “joints,” “spine”) • Date range (“January 1, 2010, to December 31, 2019”)
Thomson ONE	Private equity deals only • Screening and analysis (“private equity” and “companies and investors”) • Search entity type (“company”) • View in currency (“USD – US dollar”) • Search currency (“USD – US dollar”) • Entities involved in (“all private equity deals”) • Business description (“orthopaedics”) • Portfolio status (“currently PE/VC backed” and “formerly PE/VC backed”) • Real estate properties (“exclude”) • Disclosed valuations only? (“no”) • Investment date (“January 1, 2010, to December 31, 2019”) • Equity amount (mil) (“all”) • Investment security type (“primary”) • Previous investor (“all”) • Total equity invested by fund (mil) (“all”) All deals (PE and non-PE) • Screening and analysis (“deals and league tables” and “M and A”) • Date effective/unconditional (“January 1, 2010, to December 31, 2019”) • Target nation (code) (“United States of America”) • Target industry SIC (“801”) • Target business description (“orthopaedics”)
Zephyr	• Deal type (“acquisition”) • Period (“January 1, 2010, to December 31, 2019,” “completed-confirmed”) • Target address country (“The United States”) • US SIC (“801 – offices and clinics of doctors of medicine”) • Business description (“orthopaedics,” “joint,” and “spine”)

SIC = Standard Industrial Classification, VC = Venture Capitol

Deals meeting the selection criteria were compiled into a list. Duplicate results and deals not meeting the selection criteria were removed. The date of deal closure, buyer information (name, company type, location of headquarters, and website), seller information (name, company-type orthopaedics, orthopaedics or orthopaedic services, location of headquarters, and website), and the monetary value of the acquisition (if available) were recorded for each deal. These details were corroborated manually searching for business news articles obtained from Google searches and ensuring each practice acquired primarily provided orthopaedic care.

Buyers were classified as ASCs, solo practices, single-facility group practices, multiple-facility group practices, hospital systems, or nonhealthcare entities (eg, device companies, holding companies, and PE firms). If the buyer was a PE firm or a company receiving financial backing for the acquisition from a PE firm, the name and headquarters location of the PE firm were recorded. Acquired practices were classified as orthopaedic ASCs, solo practices, single-facility group practices, multiple-facility group practices, or orthopaedic hospitals.

Map charts of buyers and sellers by US state were generated in MapChart (https://mapchart.net/). All other graphs were generated in Microsoft Excel (version 1908).

## Results

### Historical Trends

**Figure 1:**
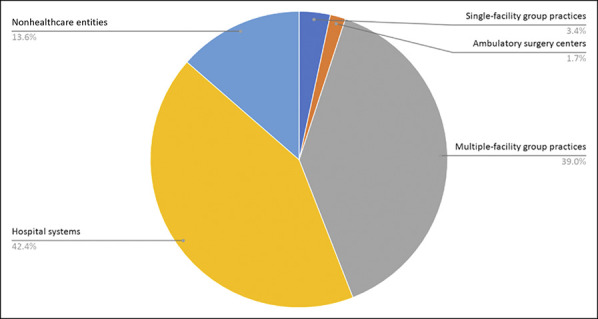
Pie chart showing composition of orthopaedic practice buyers from 2010 to 2019.

The database searches yielded 68 deals that met the selection criteria. An average of seven deals per year (SD = 3) from 2010 to 2019 was observed. The rate of acquisitions did not markedly increase or decrease between 2010 and 2019, and considerable variability was observed in the number of completed acquisitions each year (Figure 1). Acquisitions peaked in 2018 with 11 deals completed in that year. Half of the decade's acquisitions were completed by 2015.

### Buyers and Acquired Practices

**Figure 2:**
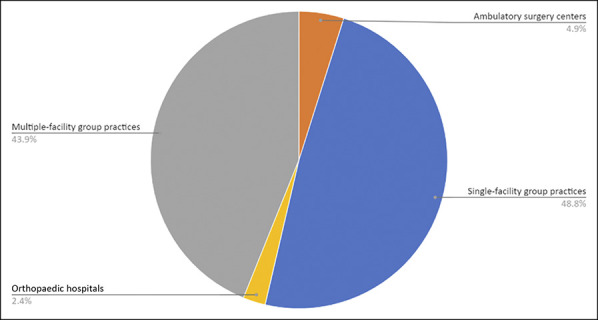
Pie chart showing composition of orthopaedic practices acquired from 2010 to 2019.

**Figure 3:**
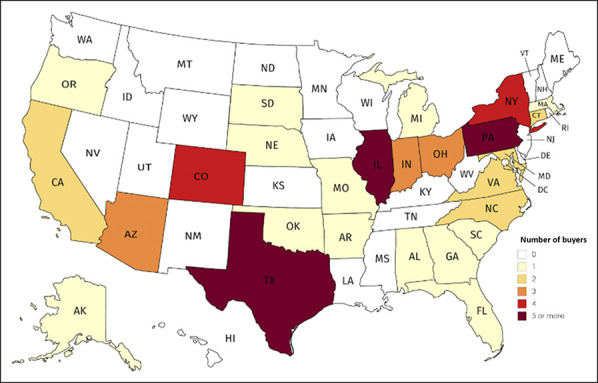
Map showing orthopaedic practice buyers by US state from 2010 to 2019.

The acquisitions involved 59 unique orthopaedic practice buyers and 68 acquired practices. Of the buyers, 42.4% were hospital systems and 39.0% were multiple-facility group practices (Figure 2). Nonhealthcare entities made up 13.6% of buyers and included five PE firms, three holding companies, and one prosthetics company. Of the acquired practices, 48.8% were single-facility group practices and 43.9% were multiple-facility group practices (Figure 3). None of the buyers or acquired practices were solo practices, and all were headquartered in the United States.

### Geographic Trends

**Figure 4:**
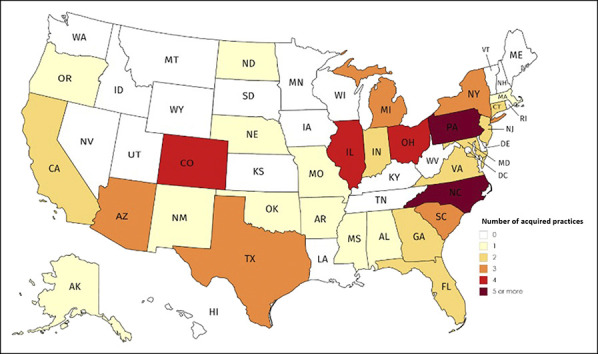
Map showing orthopaedic practice acquisitions by US state from 2010 to 2019.

**Figure 5:**
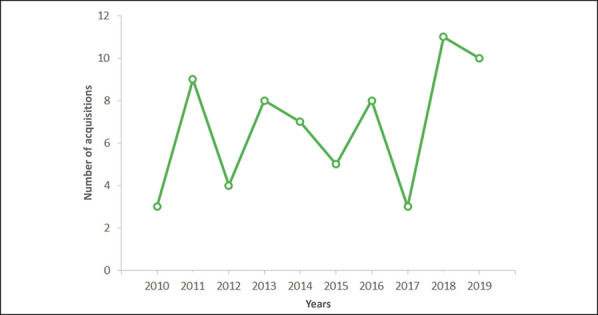
Graph showing US orthopaedic practices acquired each year from 2010 to 2019.

Most acquisitions (73.5%) involved a buyer and acquired practices that were headquartered in the same state. Buyers tended to be located along the East Coast, in the midwest, or in the southwest (Figure 4). The states with the most buyers were Pennsylvania (16.9%), Illinois (10.2%), and Texas (10.2%). Acquired clinics tended to be located along the East Coast, in the midwest, or in the southwest (Figure 5). The states with the most acquired clinics were Pennsylvania (13.2%) and North Carolina (7.4%).

### Financial and Private Equity Trends

Only four acquisitions had reported transaction values, of which three were valued at less than $5 million. The lowest reported value was $2.52 million for the 2014 acquisition of the Orthopaedic Surgery Center of Asheville, an ASC located in North Carolina, by Global Medical REIT Inc, a real estate investment trust company headquartered in Maryland. The highest reported value was $35 million for the 2011 acquisition of Orthopaedics PA, a group practice located in Arkansas, by the St. Edward Mercy Health System, a hospital system also headquartered in Arkansas.

Only five acquisitions were conducted or backed by a PE firm. These firms include Audax Group LP, Revelstoke Capital Partners LLC, Atlantic Street Capital Management LLC, MedProperties Holdings LLC, and Trive Capital Management LLC. Audax Group LP formed an acquisition vehicle, Center for Orthopedic & Research Excellence SPV, for its 2011 acquisition of the CORE Institute, a physician group headquartered in Arizona. By contrast, the latter four firms directly purchased their acquisition targets without forming an acquisition vehicle.

## Discussion

### The Landscape of Orthopaedic Practice Acquisitions

Most acquisitions of orthopaedic practices in the United States between 2010 and 2019 involved larger healthcare entities (ie, hospital systems and multiple-facility group practices), purchasing smaller group practices located in their own state. A minority of these acquisitions involved PE firms, which suggests that consolidation of orthopaedic practices is primarily driven by larger healthcare entities. These results are also consistent with previous research suggesting that physician practice consolidation is a “piecemeal” process driven by acquisitions of small practices rather than mergers or acquisitions of larger group practices and/or hospital systems.^11,12^

Intrastate acquisitions may be more common than interstate acquisitions because the former allow buyers to establish greater regional control over orthopaedic services. These types of acquisitions allow regional health systems and practices to reduce competition from other orthopaedic clinics and thereby capture a larger share of the local patient population. Furthermore, acquisitions within the same state may be more legally feasible because the purchased clinics will operate under the same state regulatory framework as the buying company. Given that physician practice acquisitions are subject to a greater regulatory burden than other corporate acquisition transactions, acquiring in-state clinics may be more legally feasible for buyers.^13^

Given that only 4 of the 68 deals included in the analysis had publicly disclosed transaction values, no conclusions can be drawn about financial trends in orthopaedic practice acquisitions from 2010 to 2019. However, it should be noted that three of the deals were valued at less than $5 million while one deal was valued at $35 million, which raises the possibility of a left-skewed distribution of deal values. A number of factors affect the valuation of a physician practice, including historical revenue, tangible assets, and payer mix, and it is reasonable to assume that at least some of these factors also influence the valuation of orthopaedic practices.^14^ Determining which factors play the largest role in the valuation of these practices could help predict future trends in orthopaedic practice acquisitions and inform the decisions of buyers and sellers involved in these transactions.

### Acquisition Trends in Nonorthopaedic Specialties

Although acquisitions of private practices have only recently gained popularity in orthopaedics, acquisition in other specialties such as dermatology and ophthalmology has increased steadily over the past decade. A 2020 study of eye care practice acquisitions by Chen et al^15^ found a total of 228 PE acquisitions of ophthalmology and optometry practices in the United States between 2012 and 2019 with notable growth in the past few years. Although 42 deals were done between 2012 and 2016, 186 deals were done from 2017 to 2019. These acquisitions, which were conducted by 29 PE-backed platform companies, included 1466 clinical locations and 2146 ophthalmologists and optometrists. Geographically, these deals took place in 40 different states, but the most popular were New York and California. The median holding period per acquired asset was 3.5 years.

A 2019 study of practice acquisitions in skin care by Tan et al^3^ found a total of 184 acquisitions of dermatology practices between May 1, 2012, and May 22, 2018, by 17 PE-backed dermatology management groups. This included 381 physical dermatology clinics at the time of acquisition. Notable growth was seen in acquisition numbers during the study period. Although there were only five acquisitions in 2012, there were 59 acquisitions in 2017 and 34 acquisitions in just the first 5 months of 2018. Geographically, these deals took place in 30 different states, but the most popular were Texas and Florida, which made up 36% of total acquired clinical locations.

### Effect of Physician Practice Acquisitions

Acquisitions of physician practices have been associated with notable effects on healthcare delivery and costs. Practice acquisitions intended to increase economies of scale and efficiency of healthcare delivery may actually increase costs for patients. According to a 2018 study by Capps et al,^16^ prices for medical services provided by physicians at an acquired practice increased by an average of 14.1% compared with preacquisition prices. The increased healthcare costs after physician practice acquisition are especially paradoxical considering that practice consolidation increases bargaining power with insurance companies and decreases the cost of healthcare delivery. For example, physician practices acquired by hospitals participating in the 340B Drug Pricing Program, which requires manufacturers to offer markedly discounted prices on outpatient drugs, benefit greatly from being able to attain drugs at much lower prices. Interestingly, a study done in 2015 found that hospitals participating in the 340B program were markedly more likely to acquire independent physician practices between 2009 and 2013 than nonparticipating hospitals.^17^

On a positive note, consolidation of multiple practices under a larger healthcare entity fosters integration of electronic health records data, although this does not necessarily lead to notable clinical integration.^18^ PE-backed acquisitions of physician practices may pose unique risks to healthcare access because of misaligned incentives. Based on trends observed in other industries with notable levels of PE investment, PE-backed practice acquisitions may exacerbate market consolidation and decrease access to care, push recently acquired practices into bankruptcy, and create legal conflicts with existing healthcare regulation such as the Stark Law and Anti-Kickback Statute.^2^ Although a lengthier discussion of the positive and negative effects of physician practice acquisitions is beyond the scope of this study, it is important to understand that these transactions can and do have a notable effect on physicians' livelihoods and patient outcomes.

### COVID-19 and the Future of Acquisitions

Throughout the course of doing this study, the advent of the COVID-19 pandemic led to notable economic turmoil, overburdening of the healthcare systems, and uncertainty for the future of small business, including private orthopaedic practices. Given this uncertainty and current lack of capital, many acquisitions of small private medical practices have come to a halt, fallen through, or slowed markedly.^19^ Despite the pandemic, there have been five public PE acquisitions in orthopaedic practices, and even more in ophthalmology in 2020.^20,21^ Furthermore, the overall trend of acquisitions may return at a higher rate than previous as many small private orthopaedic groups who are looking for a financial buffer after struggling following bans on elective procedures and social distancing guidelines limiting office volume.^22^ Ultimately, the evolving containment and regulations regarding COVID-19 in the United States remain unpredictable. Thus, the expected return of financial partnerships of PE groups and private orthopaedic practices remains uncertain.

### Limitations

The validity of our results depends on the accuracy and completeness of the transaction data obtained from the four databases. As mentioned in “Methods,” database information for each deal was corroborated using news articles and business reports. Nonetheless, the scope of our study was limited to publicly disclosed, completed transactions that were listed in at least one of the four databases, which necessarily excludes unlisted, confidential, or incomplete transactions. Including all transactions, potential or realized, in the analysis would provide a more complete picture of the acquisitions landscape and greater insight into the underlying motivations for acquisition and consolidation of orthopaedic practices.

Another issue is the lack of publicly available financial data for most of the acquisitions included in our study. Without detailed information about the funding sources for each transaction, it is difficult to ascertain whether a given deal is truly “PE-backed.”. It is likely that our study underestimates the degree of PE involvement in orthopaedic practice acquisitions because buyers receiving PE financing may opt to not disclose this information publicly.

It should be noted that many US clinics that provide orthopaedic services also provide medical services in other specialties. Because multispecialty practices were excluded from the analysis per our selection criteria (see “Methods”), our study did not capture this portion of the orthopaedic services market. Therefore, the acquisition trends reported in this study are generalizable to single-specialty orthopaedic clinics but not the orthopaedic services market as a whole.

### Future Studies

Future studies may aim to compare the acquisition trends observed for orthopaedic practices against trends described for other medical specialties such as dermatology and ophthalmology. Understanding the differences in business models, revenue sources, and patient demographics between specialties may help to explain their differing rates of consolidation and predict future market trends for private practices as a whole.

This study did not control for macroeconomic factors such as gross domestic product growth, inflation, unemployment, demographic changes, and regulatory changes between 2010 and 2019. Future analyses may explore how macroeconomic trends affect the acquisition landscape for private practices in orthopaedics and other specialties.

## Conclusion

Our study found that 70 orthopaedic practices were acquired in the United States between 2010 and 2019. Most practices were acquired by larger healthcare entities such as hospital systems and multispecialty surgical centers. Intrastate acquisitions were more common than interstate acquisitions. Our results suggest that consolidation of orthopaedic practices is predominantly driven by regional health systems and not PE firms.

## References

[1] Nikpay SS Richards MR Penson D: Hospital-physician consolidation accelerated in the past decade in cardiology, oncology. Health Aff Millwood. 2018;37:1123-1127. 29985694 10.1377/hlthaff.2017.1520

[2] Gondi S Song Z: Potential implications of private equity investments in health care delivery. JAMA. 2019;321:1047-1048. 30816912 10.1001/jama.2019.1077 PMC6682417

[3] Tan S Seiger K Renehan P Mostaghimi A: Trends in private equity acquisition of dermatology practices in the United States. JAMA Dermatol. 2019;155:1013-1021. 31339521 10.1001/jamadermatol.2019.1634 PMC6659155

[4] Rychlewski C Balshem D: This joint is jumping: PE firms knee-deep in orthopedic space. Forbes. https://www.forbes.com/sites/mergermarket/2019/08/14/this-joint-is-jumping-pe-firms-knee-deep-in-orthopedic-space/#4ae692d05cfd. Accessed August 14, 2019.

[5] Resneck JS: Dermatology practice consolidation fueled by private equity investment: Potential consequences for the specialty and patients. JAMA Dermatol. 2018;154:13-14. 29164229 10.1001/jamadermatol.2017.5558

[6] O'Hanlon CE Whaley CM Freund D: Medical practice consolidation and physician shared patient network size, strength, and stability. Med Care. 2019;57:680-687. 31295166 10.1097/MLR.0000000000001168

[7] Finnegan J: Another wave of mergers and acquisitions? Orthopedic practices attract private equity interest. FierceHealthcare. https://www.fiercehealthcare.com/practices/mergers-and-acquisitions-orthopedic-practices-private-equity-interest-jeff-swearingen. Accessed October 16, 2018.

[8] Swearingen J: Orthopedic practice consolidation jumps 45% in 2018. https://ryortho.com/2018/10/orthopedic-practice-consolidation-jumps-45-in-2018/. Accessed October 17, 2018.

[9] Private Equity Investment in Orthopedics. Provident Healthcare Partners. 2017. https://www.providenthp.com/wp-content/uploads/2020/03/Private-Equity-Investment-in-Orthopedics.pdf.

[10] More private equity firms are buying orthopedic practices. Here's why. Advisory Board. https://www.advisory.com/daily-briefing/2018/10/16/private-equity. Accessed October 16, 2018.

[11] O'Hanlon CE Whaley CM Freund D: Medical practice consolidation and physician shared patient network size, strength, and stability. Med Care. 2019;57:680-687. 31295166 10.1097/MLR.0000000000001168

[12] Capps C Dranove D Ody C: Physician practice consolidation driven by small acquisitions, so antitrust agencies have few tools to intervene. Health Aff (Millwood). 2017;36:1556-1563. 28874481 10.1377/hlthaff.2017.0054

[13] Eck WB: Physician Practice Acquisitions: Avoiding Legal Pitfalls. LexisNexis, 2019. https://www.lexisnexis.com/lexis-practice-advisor/the-journal/b/lpa/posts/physician-practice-acquisitions-avoiding-legal-pitfalls. Accessed April 14, 2020.

[14] Cleverley WO: Factors affecting the valuation of physician practices. Healthc Financ Manag. 1997;51:71-73. 10174788

[15] Chen EM Cox JT Begaj T Armstrong GW Khurana RN Parikh R: Private equity in ophthalmology and optometry: Analysis of acquisitions from 2012 through 2019 in the United States. Ophthalmology. 2020;127:445-455. 32067797 10.1016/j.ophtha.2020.01.007

[16] Capps C Dranove D Ody C: The effect of hospital acquisitions of physician practices on prices and spending. J Health Econ. 2018;59:139-152. 29727744 10.1016/j.jhealeco.2018.04.001

[17] Avalere White Paper: Hospital Acquisitions of Physician Practices and the 340B Program. Avalere. https://avalere.com/insights/avalere-white-paper-hospital-acquisitions-of-physician-practices-and-the-340b-program. Accessed January 12, 2020.

[18] West J Johnson G Jha AK: Trends in acquisitions of physician practices and subsequent clinical integration: A mixed methods study. J Eval Clin Pract. 2017;23:1444-1450. 28971563 10.1111/jep.12820

[19] Green A Oxman A Seghers L: Preparing for Private Equity Exits in the COVID-19 Era. McKinsey & Company. https://www.mckinsey.com/industries/private-equity-and-principal-investors/our-insights/preparing-for-private-equity-exits-in-the-covid-19-era. Accessed June 11, 2020.

[20] Stewart A.: Healthcare Transactions Continue During COVID-19 Pandemic: 4 Deals to Know. Becker’s ASC Review. https://www.beckersasc.com/asc-transactions-and-valuation-issues/healthcare-transactions-continue-during-covid-19-pandemic-4-deals-to-know.html. Accessed May 19, 2020.

[21] Dyrda L: 5 Orthopedic Practice Mergers, Acquisitions in 2020 So Far. Becker's Spine Review. https://www.beckersspine.com/orthopedic-spine-practices-improving-profits/item/48933-5-orthopedic-practice-mergers-acquisitions-in-2020-so-far.html. Accessed April 28, 2020.

[22] Coutré L: After Pandemic-Induced Delays, Healthcare Deals Should Speed Up. Modern Healthcare, https://www.modernhealthcare.com/mergers-acquisitions/after-pandemic-induced-delays-healthcare-deals-should-speed-up. Accessed June 22, 2020.

